# Antischistosomal Activity of the Terpene Nerolidol

**DOI:** 10.3390/molecules19033793

**Published:** 2014-03-24

**Authors:** Marcos P.N. Silva, George L.S. Oliveira, Rusbene B.F. de Carvalho, Damião P. de Sousa, Rivelilson M. Freitas, Pedro L.S. Pinto, Josué de Moraes

**Affiliations:** 1 Faculdade de Ciências de Guarulhos (FACIG/UNIESP), Av. Guarulhos, 1844, Guarulhos, SP 07025-000, Brazil; E-Mail: marcos.p.bio@gmail.com; 2 Programa de Pós-graduação em Ciências Farmacêuticas, Universidade Federal do Piauí, Teresina, PI 64049-550, Brazil; E-Mails: georgenota10@hotmail.com (G.L.S.O.); rivelilson@pq.cnpq.br (R.M.F.); 3 Programa de Pós-graduação em Biotecnologia, Universidade Federal do Piauí, Teresina, PI 64049-550, Brazil; E-Mail: rusbenebruno@hotmail.com; 4 Departamento de Ciências Farmacêuticas, Universidade Federal da Paraíba, João Pessoa, PB 58051-900, Brazil; E-Mail: damiao_desousa@yahoo.com.br; 5 Núcleo de Enteroparasitas, Instituto Adolfo Lutz, São Paulo, SP 01246-902, Brazil; E-Mail: pedro.luiz44@terra.com.br

**Keywords:** *Schistosoma*, schistosomiasis, nerolidol, schistosomicial activity, natural product, neglected tropical disease, *in vitro* studies, confocal laser scanning microscopy

## Abstract

Schistosomiasis is a neglected tropical disease that affects hundreds of millions of people worldwide. Since the treatment of this disease currently relies on a single drug, praziquantel, new and safe schistosomicidal agents are urgently required. Nerolidol, a sesquiterpene present in the essential oils of several plants, is found in many foods and was approved by the U.S. Food and Drug Administration. In this study we analysed the *in vitro* antiparasitic effect of nerolidol on *Schistosoma mansoni* adult worms. Nerolidol at concentrations of 31.2 and 62.5 μM reduced the worm motor activity and caused the death of all male and female schistosomes, respectively. In addition, confocal laser scanning microscopy revealed morphological alterations on the tegument of worms such as disintegration, sloughing and erosion of the surface, and a correlation between viability and tegumental damage was observed. In conclusion, nerolidol may be a promising lead compound for the development of antischistosomal natural agents.

## 1. Introduction

Schistosomiasis is a neglected tropical disease caused by parasitic flatworms of the genus *Schistosoma*. It is one of the most prevalent parasitic diseases in tropical and sub-tropical areas of the world, and is one of the leading causes of morbidity and mortality in endemic countries. As reviewed elsewhere, it is estimated that more than 200 million people have been infected and approximately 800 million, mostly children, live at risk of infection [1]. Schistosomiasis leads to a chronic, often debilitating, disease that impairs growth, development and productivity in infected individuals, and is strongly linked to extreme poverty. The disease results in about 300,000 deaths annually in sub-Saharan Africa alone and the disease burden, measured in disability-adjusted life-years, is estimated to exceed 70 million [2,3]. At least three species infect humans, namely *Schistosoma mansoni*, *S. japonicum* and *S. haematobium*. The major aetiological agent of human schistosomiasis is *S. mansoni* and the adult worms colonize the veins of the portal system and can live there for many years [4].

The treatment and control of schistosomiasis relies on a single drug, praziquantel, which has been administered to millions of people yearly since it was developed in the 1970s. However, the occurrence of praziquantel failures in the field or in the laboratory has been described [5,6]. A reliable alternative to praziquantel does not currently exist and the older drug oxamniquine is no longer manufactured. Thus, the resulting dependence on a single drug for the treatment of schistosomiasis is not sustainable, and for this reason, new and safe schistosomicidal agents are urgently required [7,8].

**Figure 1:**
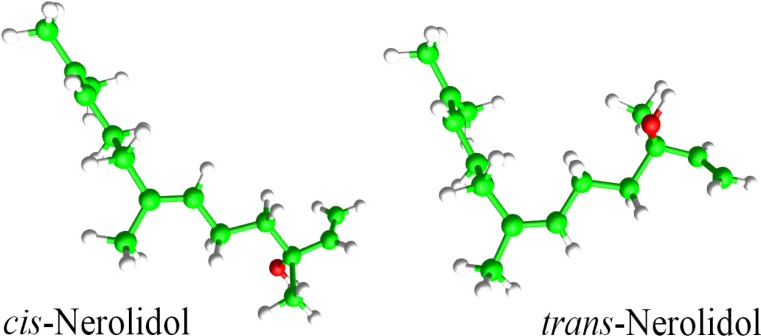
Chemical structure of nerolidol: *cis*-3,7,11-trimethyl-1,6,10-dodecatrien-3-ol and *trans*-3,7,11-trimethyl-1,6,10-dodecatrien-3-ol.

Natural products have been the basis of treatment for many human diseases [9,10]. Essential oils are highly enriched in compounds termed terpenoids that possess several biological properties such as antiparasitic activity [9,11,12]. Nerolidol (3,7,11-trimethyl-1,6,10-dodecatrien-3-ol), also known as peruviol, is an aliphatic sesquiterpene alcohol present in essential oils of several plants (Figure 1). It is frequently used in cosmetics (e.g., shampoos and perfumes) and in non-cosmetic products (e.g., detergents and cleansers) [13,14]. In medicinal fields, nerolidol has shown antioxidant [15], antinociceptive [16] and antiulcer [17] activities. Nerolidol is active against bacteria and fungi [18,19,20]. With respect to the antiparasitic effect of nerolidol, it has shown antileishmanial [21], antitrypanosomal [22] and antimalarial [23] activities as well as inhibitory effect on the growth of *Babesia* parasites [24]. On the other hand, there is little data related to the anthelmintic activity of nerolidol.

Various plant essential oils have also been investigated for use as antiparasitic agents. In this regard, the search for antischistosomal compounds from natural sources has intensified [25,26]. In the present study, we describe the antischistosomal activity of the terpene nerolidol and show its effect in the tegument of adult parasites by confocal laser scanning microscopy studies. Nerolidol was selected for this study based on the fact that this compound has well-characterised mechanisms of toxicity and is easily available and cost-effective. Additionally, nerolidol is naturally present in many foods we eat, and is approved by the U.S. Food and Drug Administration as Generally Recognized as Safe (GRAS) and was included by the Council of Europe in the list of substances granted [13,14].

## 2. Results and Discussion

Medicinal plants have been used for hundreds of years as therapeutics worldwide and the interest in natural products as new sources of antischistosomal drugs is rising. In this study, 49-day-old adult *S. mansoni* were cultured in RPMI 1640 medium in the presence of nerolidol, a sequisterpene present in essential oils of several plants.

### 2.1. Nerolidol Affected the Viability of Schistosomes

**Figure 2:**
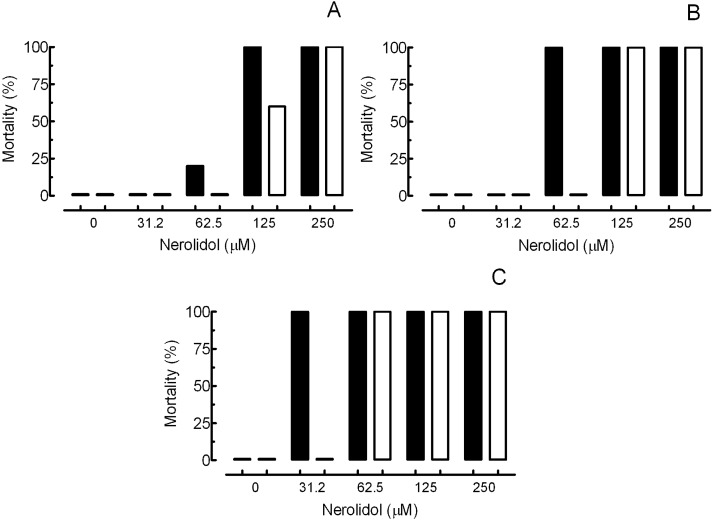
*In vitro* effect of nerolidol on the survival of *Schistosoma mansoni* after 24 h (**A**), 48 h (**B**) and 120 h (**C**) of treatment. Pairs of adult worms, males (closed bars) and females (open bars), were incubated in 24-well culture plates containing RPMI 1640 medium and treated with nerolidol at different concentrations. Mortality data are presented from ten worm couples and values correspond to the sum of the adult schistosomes obtained from three separate experiments performed in triplicate (*n* = 2) and quadruplicate (*n* = 1).

The results of the *in vitro* studies with schistosomes exposed to nerolidol at concentrations of 15.6, 31.2, 62.5, 125 and 250 μM and control groups are summarised in Figure 2 and Table 1. In the negative control group (RPMI 1640 medium containing 0.5% DMSO), schistosomes showed normal motor activity and had no observed mortality. In contrast, 3 µM praziquantel resulted in complete loss of motor activity and caused the death of all parasites. These observations in the negative and positive control groups are similar to that described in the literature [27,28,29].

During incubation with nerolidol (15.6 to 250 μM), all of the adult worm pairs were separated into individual male and female worms. Interestingly, nerolidol at 62.5 μM was lethal to 100% of male adult parasites after 48 or 72 h of exposure *in vitro*, whereas in this same time period, no mortality was observed in the female worms (Figure 2 and Table 1). However, nerolidol at 250 and 125 μM signiﬁcantly reduced motor activity in *S. mansoni* and resulted in death of 100% of male and female parasites on first and second day of incubation, respectively (Table 1). These findings show that the nerolidol has antischistosomal properties as well as indicate that adult male parasites are more susceptible to the nerolidol than female worms. A similar variation in drug susceptibility between male and female schistosomes have also been observed for praziquantel [30] and with other natural products such as volatile organic components of *Ageratum conyzoides* [31] and ginger rhizomes [32].

**Table 1:** *In vitro* effects of nerolidol against adult *Schistosoma mansoni*.

Group	Period of incubation (h)	Separated worms (%) ^a^	Dead worms (%) ^a^		Motor activity reduction (%) ^a^			
			M	F	M	F	M	F
Control ^b^	24	0	0	0	0	0	0	0
0.5% DMSO	48	0	0	0	0	0	0	0
	72	0	0	0	0	0	0	0
	96	0	0	0	0	0	0	0
	120	0	0	0	0	0	0	0
PZQ	24	0	100	100	0	0	100	100
3 µM	48	0	100	100	0	0	100	100
	72	0	100	100	0	0	100	100
	96	0	100	100	0	0	100	100
	120	0	100	100	0	0	100	100
Nerolidol	24	100	100	100	0	0	100	100
250 µM	48	100	100	100	0	0	100	100
	72	100	100	100	0	0	100	100
	96	100	100	100	0	0	100	100
	120	100	100	100	0	0	100	100
Nerolidol	24	100	100	60	0	0	100	100
125 µM	48	100	100	100	0	0	100	100
	72	100	100	100	0	0	100	100
	96	100	100	100	0	0	100	100
	120	100	100	100	0	0	100	100
Nerolidol	24	100	20	0	0	60	100	0
62.5 µM	48	100	100	0	0	40	100	60
	72	100	100	0	0	0	100	100
	96	100	100	100	0	0	100	100
	120	100	100	100	0	0	100	100
Nerolidol	24	100	0	0	0	0	0	0
31.2 µM	48	100	0	0	40	0	0	0
	72	100	0	0	0	0	100	0
	96	100	100	0	0	100	100	0
	120	100	100	0	0	100	100	0
Nerolidol	24	100	0	0	0	0	0	0
15.6 µM	48	100	0	0	0	0	0	0
	72	100	0	0	0	0	0	0
	96	100	0	0	0	0	0	0
	120	100	0	0	0	0	0	0

^a^ Percentages relative to the 20 worms investigated. Values correspond to the sum of the adult schistosomes obtained from three separate experiments performed in triplicate (*n* = 2) and quadruplicate (*n* = 1). Male parasite (M). Female parasite (F); ^b^ RPMI 1640 medium.

### 2.2. Nerolidol Caused Tegumental Damage in Schistosomes

**Figure 3:**
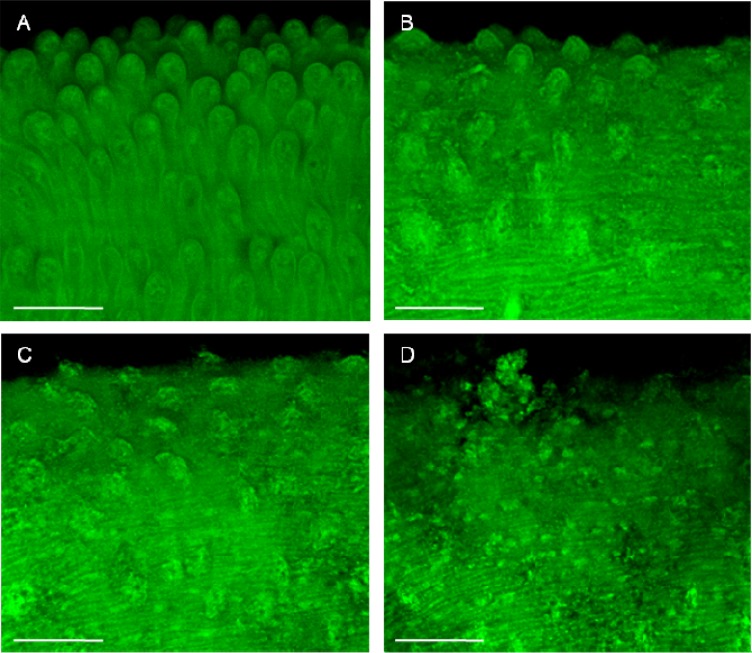
Microscopy investigation of *Schistosoma mansoni* male worm after *in vitro* incubation with nerolidol. After 5 days or in the case of death, schistosomes were fixed and monitored using a confocal microscopy. (**A**) Negative control. (**B**) 62.5 µM nerolidol. (**C**) 125 µM nerolidol. (**D**) 250 µM nerolidol. Scale bars = 50 µm.

The schistosome worm is covered by a syncytial cytoplasmic layer, the tegument, which is of crucial importance for parasite survival [33]. Thus, the worm tegument is considered an important drug target in schistosomiasis [26]. We used confocal laser scanning microscopy as a tool to evaluate whether the exposure to nerolidol could affect the tegument of *S. mansoni* adult worms. The morphological features of adult *S. mansoni* in the control groups were in agreement with previous reports [27,34,35]. As can be observed in Figure 3, no abnormality was noticed in *S. mansoni* adult worms in the negative control group and, thus, the dorsal tegumental surface of male worms showed intact tubercles (Figure 3A). In contrast, nerolidol at 62.5 to 250 µM caused morphological alterations in the tegument of parasites. For example, slight tegumental damage was observed in the schistosomes treated with nerolidol at 62.5 µM (Figure 3B), whereas extensive tegumental damages were observed at 125 and 250 µM (Figure 3C,D). The principal alterations in *S. mansoni* male worms exposed to nerolidol were disintegrated tubercles as well as sloughing and erosion of the surface.

**Figure 4:**
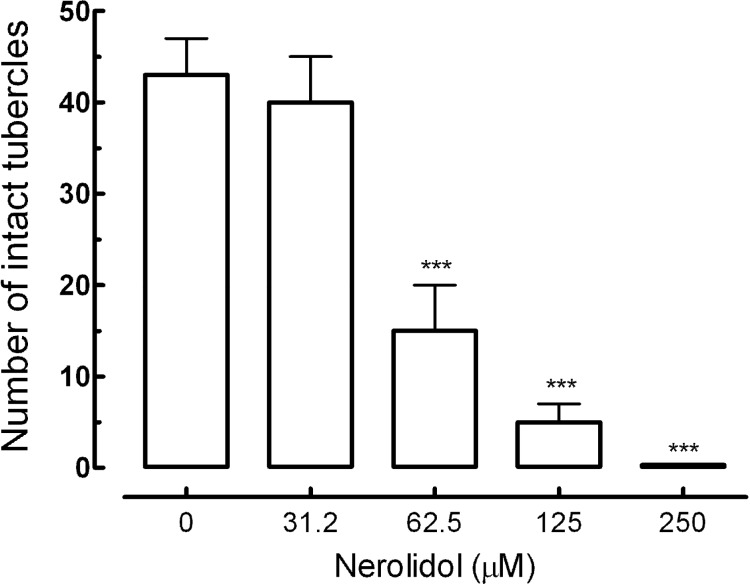
Morphological changes on the tegument of *S. mansoni* male worms after treatment with nerolidol. Quantitative analysis, measured in a 20,000 µm^2^ of area in a dorsal region of male parasite, was performed using three-dimensional images obtained from confocal microscope (see Figure 3). A minimum of three tegument areas of each parasite were assessed. Values are means ± SD (bars) of ten male adult worms. *** *p* < 0.001 compared with untreated groups.

Additionally, we performed a quantitative analysis to observe tegumental damage on *S. mansoni* male worm. In this case, areas of 20,000 µm^2 ^ of the tegument of schistosomes were assessed, and the number of tubercles was counted. Nerolidol caused changes in the tubercles of schistosomes in a concentration-dependent manner. As can be observed in Figure 4, the number of normal tubercles on schistosomes of the control group was 43 (± 4), while in the parasites treated with 62.5 µM of nerolidol, the number was 15 (± 5). In addition, when the concentration of nerolidol was increased to 250 μM, no intact tubercles were seen.

A recent study reported that nerolidol (10 to 100 μM) did not show any antischistosomal activity against *S. mansoni* adult worms [36]. However, in our hands, nerolidol exhibited antischistosomal properties at lower concentrations (31.2 and 62.5 µM). It has been demonstrated that different *Schistosoma* strains exhibit different drug sensitivity patterns [37] and thus we attribute the differences between our results and previous data to the use of different *S. mansoni* strains. Moreover, the researchers used *trans*-nerolidol [36], whereas we used a racemic mixture; therefore, it is possible that *trans*-nerolidol is the far less active isomer. Indeed, the results in the present study showed that the nerolidol has antischistosomal properties as well as revealed that nerolidol induced severe tegumental damage in adult schistosomes. Additionally, quantitative analysis showed that nerolidol caused alterations on the tubercles of male parasites in a concentration-dependent manner.

The mechanism by which nerolidol exerts its *in vitro* schistosomicidal effect is not clear. However, a correlation between viability and tegumental damage was observed (Table 1, Figure 2, Figure 3 and Figure 4). Due to its inherent lipophilicity, nerolidol will easily cross plasmatic membranes [19] and, consequently, may also interact with intracellular molecules of parasites. Comparable results were obtained by previous works using other antischistosomal natural compounds, such as epiisopiloturine [38], (+)-limonene epoxide [39], dermaseptin [40] and phytol [41].

## 3. Experimental

### 3.1. Drugs

Nerolidol (a mixture of *cis*- and *trans*-nerolidol) (Figure 1) was purchased from Sigma-Aldrich (St. Louis, MO, USA) and praziquantel was purchased from Merck (São Paulo, SP, Brazil). Stock solutions (8 mM nerolidol and 4 mM praziquantel) were prepared in dimethyl sulfoxide (DMSO, Sigma-Aldrich) and were used for *in vitro* experiments.

### 3.2. Parasite

*Schistosoma mansoni* (BH strain) was used in this study. Schistosomes were obtained from experimentally infected *Mesocricetus auratus* hamsters as described previously [26,34]. Animals were subcutaneously infected with approximately 150 cercariae following standard procedures of our laboratory [26]. Seven weeks post-infection, adult *S. mansoni* were removed from the hepatic portal system and mesenteric veins and cultured in RPMI 1640 culture medium supplemented with 200 IU/mL penicillin and 200 µg/mL streptomycin (Invitrogen, São Paulo, SP, Brazil) 10% and foetal bovine serum at 37 °C in an atmosphere of 5% CO_2_ until use.

### 3.3. In Vitro Antischistosomal Assay

*For* the *in vitro* test with *S. mansoni*, parasites were incubated in a 24-well culture plate (TPP, St. Louis, MO, USA), placing one coupled worm pair in each well, containing the RPMI 1640 medium at 37 °C in a 5% CO_2_ atmosphere [27,35]. Nerolidol was used at concentrations of 15.6 to 250 μM (15.6, 31.2, 62.5, 125 and 250 μM) in culture plates with a ﬁnal volume of 2 mL. The parasites were kept for 120 h and monitored every 24 h using an inverted microscope. The effect of the drug was assessed with emphasis on changes in worm motor activity and alteration in the tegument as previously described [41]. Death was deﬁned as no movement observed for at least 1 to 2 min of examination [26,42]. In addition, worms were prepared for confocal laser scanning microscopy examination whose details are described below. The control worms were assayed in RPMI 1640 medium as a negative control group and 3 μM praziquantel as a positive control group.

### 3.4. Microscopy Studies

To observe morphological alterations on the tegument of schistosomes after *in vitro* assays, male worms were monitored using a confocal laser scanning microscope as described elsewhere [26]. The parasites were fixed in a formalin-acetic acid- alcohol solution (FAA) and analysed under a confocal laser scanning microscope (LSM 510 META, Carl Zeiss, Standorf Göttingen, Vertrieb, Germany). Autofluorescence was excited with a 488 nm line from an Argon laser, and emitted light was collected at 505 nm [43].

To assess the damage in the tegument of *S. mansoni* as a quantitative method, areas (20,000 µm^2^) of the dorsal surface of male worms were assessed, and the numbers of tubercles were counted using three-dimensional images obtained from confocal microscopy according to standard procedures [26]. The area was calculated using LSM Image Browser software (Zeiss).

## 4. Conclusions

The results of the present study show that a racemic mixture of *E*- and *Z*-nerolidol possesses *in vitro* antischistosomal activity against *Schistosoma mansoni* adult worms. This terpene decreased the motor activity and caused the death of worms; additionally, nerolidol was able to cause morphological alterations in the tegument of adult schistosomes. In general, our results are important as there is an urgent need to develop new agents against schistosomiasis. Since nerolidol is generally recognized as safe, this compound may have antiparasitic applications. Further studies are necessary to elucidate mechanisms of action of nerolidol as well as to examine the *in vivo* effects of this natural compound in *S. mansoni*-infected animals. 
